# Eye-tracking reveals agency in assisted autistic communication

**DOI:** 10.1038/s41598-020-64553-9

**Published:** 2020-05-12

**Authors:** Vikram K. Jaswal, Allison Wayne, Hudson Golino

**Affiliations:** Department of Psychology, University of Virginia, Charlottesville, VA USA

**Keywords:** Human behaviour, Autism spectrum disorders

## Abstract

About one-third of autistic people have limited ability to use speech. Some have learned to communicate by pointing to letters of the alphabet. But this method is controversial because it requires the assistance of another person—someone who holds a letterboard in front of users and so could theoretically cue them to point to particular letters. Indeed, some scientists have dismissed the possibility that any nonspeaking autistic person who communicates with assistance could be conveying their own thoughts. In the study reported here, we used head-mounted eye-tracking to investigate communicative agency in a sample of nine nonspeaking autistic letterboard users. We measured the speed and accuracy with which they looked at and pointed to letters as they responded to novel questions. Participants pointed to about one letter per second, rarely made spelling errors, and visually fixated most letters about half a second before pointing to them. Additionally, their response times reflected planning and production processes characteristic of fluent spelling in non-autistic typists. These findings render a cueing account of participants’ performance unlikely: The speed, accuracy, timing, and visual fixation patterns suggest that participants pointed to letters they selected themselves, not letters they were directed to by the assistant. The blanket dismissal of assisted autistic communication is therefore unwarranted.

## Introduction

Communication—the sharing of information, beliefs, and desires with other individuals—is so essential to well-being that it is considered a fundamental human right^1^. About 30% of autistic children and adults have limited ability to communicate using speech^2^, and most are never provided access to an effective alternative language-based means of communication^3^. Not having a way to communicate effectively using language is arguably the most significant aspect of their disability, severely limiting educational, social, and employment opportunities. However, some nonspeaking autistic people have learned to communicate by typing on a keyboard or pointing to letters printed on a letterboard^4^. These methods have enabled some individuals to graduate from college^5^, to write acclaimed poetry and essays^6^, and to publish a best-selling memoir^7^. But they have also generated controversy.

Most nonspeaking autistic people who communicate in these ways require assistance from another person—someone who physically supports their typing finger or wrist, for example, or someone who holds a letterboard in front of them as they independently point to letters. According to practitioners and users, the assistant helps to mitigate sensory, motor, attentional, and self-regulatory challenges many nonspeaking autistic people face^8^. Indeed, a large body of empirical research has shown that many autistic people have significant difficulty with motor coordination^9^, and hyper- or hypo-reactivity to sensory input is so common in autism that it is one of the symptoms included in the diagnostic criteria^10^. Due to the heterogeneous and developmental nature of sensory motor differences in autism, it is widely recognized that different autistic individuals require different kinds and levels of support at different points in their lives^11^.

But in the case of assisted communication in autism, the assistant’s participation in the process has raised doubts about whose thoughts are being conveyed. Studies with nonspeaking autistic people who type while an assistant supports their hand or arm have shown that the text they compose can be influenced by the assistant: If the typist and the assistant are shown different images, for example, the typist rarely types the name of the image they were shown and may instead type the name of the image the assistant was shown^12,13^. The results of these experimental “message passing” tests have led many scientists to conclude that anyone who appears to communicate with assistance—including individuals with accomplishments like those described above—is actually responding to subtle cues from the assistant^14,15^. As a result, the American Speech-Language-Hearing Association recently published two position statements actively discouraging speech-language pathologists from teaching individuals to type or point to letters with assistance and cautioning against believing information obtained from individuals who communicate in these ways^16,17^.

Yet behavioural scientists have shown repeatedly that tests that fail to take into account a group’s unique developmental history can underestimate or misrepresent the abilities of members of that group^18–21^. Nonspeaking autistic people have very different experiences with communication (and many other things) from most other people^22^. For example, children who can talk receive years of prompting and feedback from adults on how to report information their interlocutor does not know^23^, the essence of a message passing test. Nonspeaking children, who do not have an effective way to express themselves using language, do not receive this training in how to report information someone else does not know. Even when some individuals begin to learn to type or point to letters with assistance, the training focuses on content known to the assistant^24^. These kinds of differences in experience, combined with an unfamiliar experimental setting^25^ and elevated levels of anxiety common in autism^26^, may explain some nonspeaking autistic people’s difficulty with message passing tests. They highlight the need for alternative approaches for investigating whether it is possible for individuals who communicate with assistance to convey their own thoughts^27^.

We report here a study designed to quantitatively characterize experienced letterboard users’ communication *in situ*. Rather than assessing users’ performance on experimental message passing tests, we used head-mounted eye-tracking to measure how quickly and accurately they looked at and pointed to letters as they participated in a familiar activity: responding to questions about a piece of text, a common instructional practice at the educational centre where data collection took place. Head-mounted eye-tracking has been used to study a variety of complex skills *in situ*, from walking to driving to piano playing. Investigating the coordination between individuals’ gaze and movements can provide insight into how they plan, coordinate, and execute those movements, and it can inform inferences about underlying cognitive processes^28^.

The study reported here builds on a provocative finding described in a case study of a nonspeaking autistic participant who typed while his hand was physically supported by an assistant^27^. Eye-tracking showed that the participant looked at letters before typing them more often than would be expected by chance, although difficulties in calibrating the eye-tracker yielded limited data that the authors described as “full of noise.” In the current work, we investigated communicative agency using more robust eye-tracking technology, additional quantitative analyses, and a sample of nine nonspeaking autistic individuals who communicate without receiving physical support. That is, unlike the participant in the case study, the participants in the current work point to letters independently; an assistant holds a letterboard in front of them but does not touch them.

If the assistant signals to letterboard users which letters to select—by moving the letterboard slightly, for example^29^—the speed and accuracy with which users look at and point to letters should be limited by two factors. First, psychologists have long known that how quickly and accurately someone can respond to a cue depends on the salience of the cue and the number of cue-response alternatives^30^. On a cueing account of a letterboard user’s performance, the assistant would need to deliver a cue that identified which of 26 letters to point to, and the user would need to detect, decode, and act upon that cue. Each of these steps would take time and would be subject to error, especially given the subtlety of the cues the assistant is hypothesized to deliver and the 26 cue-response alternatives.

Second, on a cueing account, the deliver-detect-decode-act sequence would have to be repeated for each letter in a response. For example, for a letterboard user to spell the word “sunflower,” the assistant would first deliver a cue signalling “s,” which the user would then detect, decode, and act upon; the assistant would then cue “u,” and the user would detect, decode, and act upon that cue; and so on for each letter in the word (and for each letter in each word in a multi-word response). Thus, slow or inaccurate looking and pointing to letters would be consistent with a cueing account of users’ performance; fast and accurate looking and pointing would not. To foreshadow our results, users not only looked at and pointed to letters quickly and accurately even in lengthy responses, but patterns in their response times and visual fixations revealed planning and production processes suggesting that they were conveying their own thoughts.

**Figure 1:**
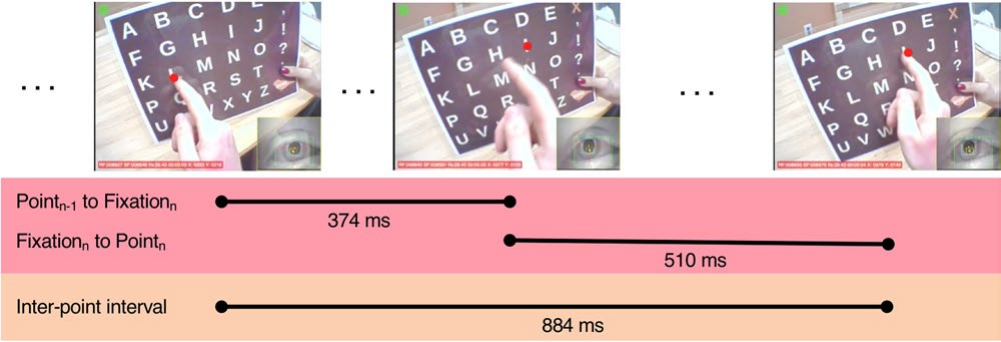
Quantitative characterization of *in situ* letterboard use. Participants wore eye-tracking glasses that provided a video record of their field of view and their right eye’s movements. Shown here are frames from Participant 2’s processed video (produced using Yarbus software, version 2.5.0, www.positivescience.com) as he pointed to the first two letters in the word “like” in the response available in Supplementary Video 1. The red circle shows where the eye-tracker estimated he was looking; the circle was not visible during his session (see Methods). The left frame shows the end of his point to the letter “L”; the position of the red circle indicates he was also looking at “L” at that time. The middle frame shows that 374 ms later, he began fixating the letter “I” as his finger was moving toward it. The right frame shows that 510 ms after that, the participant began pointing to the letter “I” (and he continued to fixate the letter “I”). The time between the end of the point to the letter “L” and the beginning of the point to the letter “I” (the inter-point interval) was 884 ms. Ellipses represent frames not shown in the figure.

Participants were nine nonspeaking autistic young adults who had been learning to use a letterboard for at least 2.25 years (see Supplementary Table S1 for descriptive characteristics of the sample). They wore a head-mounted eye-tracker as they responded to 24 questions about an article read aloud to them by a familiar assistant (see the Methods section for details). Participants composed their responses by pointing independently with the index finger of their right hand to letters on a letterboard held vertically by the assistant; the assistant did not touch them. The same individual served as the assistant for all participants. Responses ranged in length from a single word (e.g., Question: Name a type of flower; Answer: “Sunflower”) to over a dozen words (e.g., Question: Can you think of something you have to wait for? Answer: “That is hard. I feel like world is waiting on me not the other way around”). As Fig. 1 shows, the eye-tracker provided a video record of each session, allowing for off-line coding of which letters participants pointed to and where they looked. Supplementary Videos 1–3 show an example response from three participants.

## Results

Analyses were conducted using *R*^31^ under the *RStudio* environment^32^.

### Word and letter accuracy

If a word was spelled using the exact letters required in the correct order, it was coded as correct. If a word was spelled using too few or too many letters between its first and last letters, if it was missing its first or last letter, or if it was interrupted by the assistant resetting the letterboard, it was coded as incorrect. Words that included incorrect leading or trailing letters but were otherwise spelled correctly could have represented the abandoned start of a different word rather than a misspelling of the identified word; they were therefore coded as correct (e.g., one participant began a response by pointing first to the letter “c” and then spelling “explored”). Most of the 44 to 84 words participants produced during their sessions were spelled correctly (*M*: 83% correct, *range*: 71–92%).

To calculate letter accuracy, we analysed the number of letters pointed to that were correct in the context of the words ultimately spelled. Almost all of the 277 to 435 letters participants pointed to were correct in the context of the words produced (*M*: 94% correct, *range*: 88–97%). Supplementary Table S2 shows the word and letter accuracy data for each participant.

Participants did not achieve this high level of accuracy by looking to the assistant for explicit guidance about which letters to point to: They looked at the letterboard for over 90% of the time they were spelling (*range*: 92–99%), and six of the nine participants never looked at the assistant at all while they were spelling (see Supplementary Table S3).

### Inter-Point interval (IPI)

**Figure 2:**
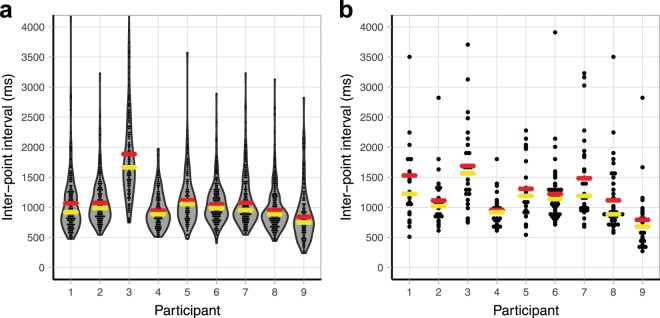
Inter-point interval (IPI). (**a**) IPI between letters within correctly spelled words. (6 observations of 1976 total were slower than 4250 ms and are not shown.) (**b**) IPI between letters in each participant’s longest sequence of points to correct letters within a response. (1 observation of 252 total was slower than 4250 ms and is not shown.) Each dot represents an individual datapoint. Yellow lines show the medians, and red lines show the means.

The speed and fluency with which participants spelled suggest that they were not relying on subtle cues from the assistant either. We calculated the inter-point interval (IPI)—the time between the end of a point to one letter and when the finger made contact with the next letter. Within correctly spelled words (*M*_word length_: 4.80 letters, *range*: 2–13), the median IPI was 952 ms (Fig. 2a); within participants’ longest string of consecutive points to correct letters in a single response (*M*_string length_: 29 letters, *range*: 22–47), it was 1054 ms (Fig. 2b). In other words, participants pointed to the next correct letter (from 26 possible letters) about one second after their finger left the previous letter, and each participant produced at least one response where they rapidly pointed to over 20 correct letters in a row. Given the 26 cue-response alternatives a cueing account would entail, it is unlikely that they could have achieved this level of fluency by responding to subtle cues from the assistant.

**Figure 3:**
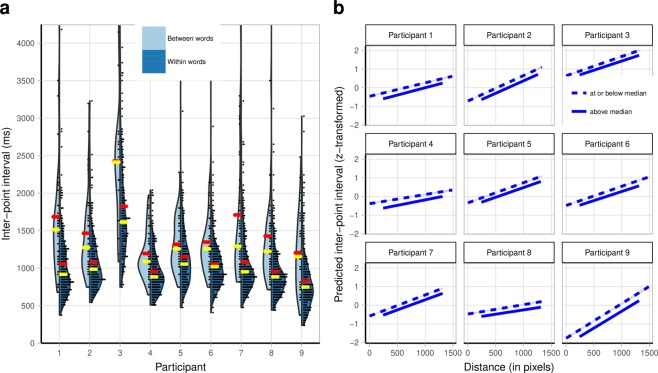
Inter-point interval (IPI) patterns. (**a**) Time between end of a point to correct letter_n-1_ and point to correct letter_n_ as a function of whether the transition from letter_n-1_ to letter_n_ crossed a word boundary (light blue) or was within a word (dark blue). Each dot represents an individual datapoint. Yellow lines show the medians, and red lines show the means. (7 of 350 between-word observations and 6 of 2417 within-word observations were slower than 4250 ms and are not shown.) (**b**) Individual model fits for each participant predicting IPI between consecutive points to correct letters within words, using as predictors the distance between those letters on the letterboard and how frequently they occur consecutively in English. Bigram frequency is shown as a median split but was a continuous variable in the linear mixed-effects analysis reported in the text. Shading represents 95% confidence intervals.

We also identified two patterns in the IPI data which, when observed in studies with non-autistic participants, have been attributed to cognitive processes underlying fluent spelling. First, non-autistic adults using a keyboard are slower to type the first letter of the second morpheme of a compound word (e.g., “b” in “sailboat”) than to type letters within either morpheme^33^. The increase in response time at the morpheme boundary is thought to reflect the time it takes typists to plan production of the second morpheme. There is no spacebar on the letterboard, so for participants in our study, a multi-word response was analogous to a compound word. We found that participants were significantly slower to point to the first letter of a new word in a multi-word response than to point to letters within words (Fig. 3a), suggesting they engaged in a similar planning process prior to the production of new words, *b* = 0.75, SE = 0.07, *t*(7.96) = 10.76, *p* <0.0001, 95% CI [0.59, 0.89] (see Supplementary Notes for model details).

Second, non-autistic adults type pairs of letters that co-occur frequently faster than pairs of letters that co-occur infrequently, which is thought to reflect the internalization of orthographic regularities in the typed language^34^. In a linear mixed-effects model, in which we controlled for the physical distance between the two letters on the letterboard, we found that participants were significantly faster to point to the second letter in more frequent compared to less frequent pairs of letters in English (Fig. 3b), β = −0.18, SE = 0.02, *t*(8.66) = 9.25, *p* < 0.0001, 95% CI [−0.23, −0.14] (see Supplementary Notes for model details). For example, “n” and “t” are diagonally adjacent on the letterboard (see Fig. 1), and so “nt” and “tn” are necessarily the same physical distance apart. However, “nt” is much more common within English words than “tn”^35^, and this results in a shorter IPI for “nt” than “tn.” Participants in our study had been exposed to printed English words throughout their lives and had at least 2.25 years of experience using a letterboard. These IPI patterns suggest that, like non-autistic individuals, they drew on internalized English orthographic regularities when spelling.

### Anticipatory fixations

The accuracy, speed, and IPI patterns described so far are consistent with what would be expected if participants were conveying their own thoughts. But it is possible that participants passively extended their finger as the assistant rapidly moved the letterboard beneath it to each letter^36^. To investigate this possibility, we analysed how often and by how long participants visually fixated a correct letter in a response before their finger came into contact with it. The criterion for a fixation was a look to a letter lasting at least 99 consecutive ms^37^. As an example of how anticipatory fixations were identified, when Participant 3 was asked to name a type of flower, he responded “sunflower (done)”. We asked whether he fixated “s” after the letterboard had been placed and before “s” was pointed to, whether he fixated “u” after pointing to “s” and before “u” was pointed to, and so on. Occasionally, a fixation of the next letter in a response began before or during the point to another letter (rather than after the point had ended). These were also considered anticipatory fixations. (See Methods for additional eye-tracking details.)

**Figure 4:**
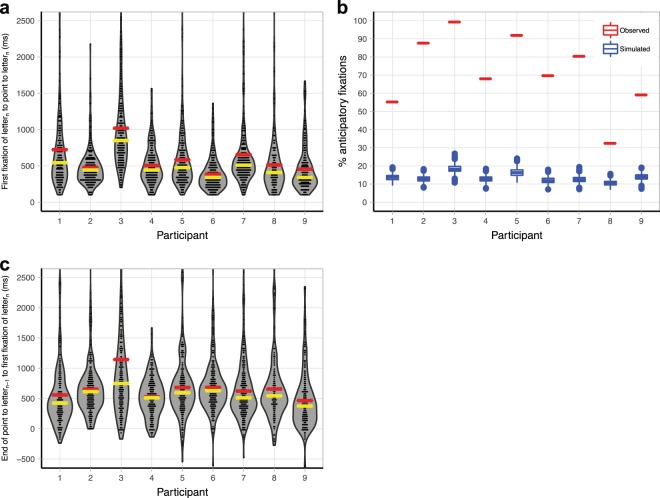
Anticipatory fixations of correct letters. (**a**) For fixated letters, time between the beginning of the first fixation of letter_n_ and the point to letter_n_. Minimum fixation length was 99 ms. (24 observations out of 2192 total were slower than 2500 ms and are not shown.) (**b**) Observed percentage of points to correct letters preceded by a fixation of that letter (red lines) vs. simulated percentage of points to correct letters preceded by a fixation of that letter if fixations had been random (blue boxplots representing 1000 simulations). (**c**) Time between the end of the point to letter_n-1_ and the beginning of the first fixation of letter_n_. (30 observations out of 2192 total were slower than 2500 ms and are not shown.) For violin plots, each dot represents an individual datapoint. Dots at or below 0 represent occasions where a participant’s fixation of letter_n_ began before or during the point to letter_n-1_ and continued after the point to letter_n-1_ had ended. Yellow lines show the medians, and red lines show the means.

Accurate, goal-directed pointing is normally visually guided^38^. If participants simply touched letters the assistant placed in front of their finger, they would not be expected to consistently fixate letters before their finger made contact with them. In fact, we found that participants fixated, on average, 71% (*range*: 32–99%) of correct letters before pointing to them (see Supplementary Table S4 for anticipatory fixation data for each participant), and fixations of correct letters preceded the points to those letters by about half a second (*median*: 476 ms; Fig. 4a).

To calculate the likelihood by chance that participants would fixate correct letters before pointing to them, we used a method similar to one described in the case study mentioned earlier^27^. For each participant, we created a list of all of the letters they fixated (correct or incorrect) before pointing to a correct letter during their session. For example, before pointing to “s” in “sunflower,” a participant might fixate “r,” “v,” “r” again, and then “s.” These four letter tokens (two tokens of “r,” one of “v,” and one of “s”) would be added to that participant’s list; their complete list would contain tokens representing all fixations made prior to pointing to correct letters in their session. Each participant’s list differed in the number of tokens because different participants fixated different numbers of letters over the course of their session (*range*: 793–1141 fixations). These lists also differed in the number of tokens of particular letters: Participants looked at some areas of the letterboard more than others, the eye-tracking calibration was better for some areas of the letterboard than others (see Supplementary Methods for calibration information), and both of these differed across participants.

We used these custom lists of anticipatory fixations to create a simulation for each participant. For each correct letter a participant pointed to, the simulation selected randomly (with replacement) one of the letter tokens from their custom list of fixations and continued selecting tokens from that list up to the total number of letters the participant actually fixated before pointing to that letter. Continuing with the “sunflower” example, for instance, if a participant fixated four letters before pointing to “s,” the simulation randomly selected four tokens from that participant’s complete list of anticipatory fixations. If one or more of the randomly selected tokens was an “s,” the “s” in “sunflower” was considered to have been fixated in the simulation. This process was repeated for each correct letter the participant pointed to. (When there were no fixations recorded in the actual data before the participant pointed to a correct letter in a word, the simulation also recorded that no fixation occurred.) We then calculated the percentage of points to correct letters that were considered to have been fixated in the simulation of a participant’s session and repeated the simulation 1,000 times for each participant. As Fig. 4b shows, these simulations showed that each participant was more likely to fixate correct letters than would be expected if they had been fixating letters randomly.

Still, it could be argued that participants fixated letters before pointing to them because they were somehow cued to those letters by the assistant rather than because participants selected the letters themselves^39^. The specificity and speed with which participants fixated correct letters make this cueing possibility unlikely. The number of fixations to locate a target is a measure of search efficiency^40^. It is unlikely that the assistant could consistently deliver cues that precisely identified which of 26 letters to fixate or that participants could consistently detect and interpret such cues without error^30^. Thus, if anticipatory fixations were driven by cues from the assistant, we expected participants to fixate several incorrect letters before the correct one. In fact, correct letters were usually the first or second item on the letterboard fixated (*M*: 60%, *range:* 48–66%; see Supplementary Table S4), and fixations of correct letters began about half a second after the point to the previous letter ended (*median*: 544 ms; Fig. 4c).

Finally, the two patterns characteristic of fluent spelling in non-autistic typists described earlier in the context of the IPI data are also evident in the fixation data. First, participants were slower to fixate the first letter of a new word in a multi-word response than to fixate letters within words, *b* = 0.41, SE = 0.09, *t*(8.69) = 4.36, *p* = .0020, 95% CI [0.20, 0.62] (see Supplementary Fig. S1). Second, they were faster to fixate the second letter in a pair of letters within a word the more frequent the pair is in English even when controlling for the physical distance between the two letters, β = −0.19, SE = 0.04, *t*(8.71) = 5.13, *p* = .0007, 95% CI [−0.27, −0.11] (see Supplementary Fig. S2). (Details of the models are provided in the Supplementary Notes).

## Discussion

**Figure 5:**
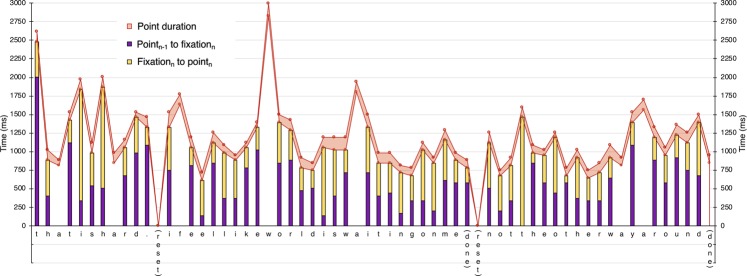
Timing of visual fixations and points in Participant 2’s response shown in Supplementary Video 1. Question: Can you think of something you have to wait for? Answer: “That is hard. (reset) I feel like world is waiting on me (done) (reset) not the other way around (done)”. Purple shading shows the time between the end of the point to letter_n-1_ (or when letterboard was placed) and the first fixation of letter_n_; yellow shading shows the time between the beginning of the first fixation of letter_n_ and the beginning of the point to letter_n_. For letters without a shaded column, coders did not code an anticipatory fixation. The lower red line shows when the participant’s pointing finger made contact with letter_n_; the upper red line shows when his finger stopped being in contact with letter_n_ (i.e., the end of the point to letter_n_). The red shading shows how long the participant’s finger remained in contact with letter_n_.

One of the challenges in understanding members of groups with very different backgrounds from the people studying them is determining what measures can provide an accurate picture of their abilities^18–21^. By quantitatively characterizing *in situ* communication in a sample of nonspeaking autistic people who have learned to use a letterboard, we have come to a very different conclusion about their ability to convey their own thoughts than has been suggested by previous research using message passing tests^12,13^. The accuracy, speed, timing, and visual fixation patterns reported here suggest that participants were not simply looking at and pointing to letters that the assistant holding the letterboard cued them to. Instead, our data—like those of the case study described earlier^27^—suggest that participants actively generated their own text, fixating and pointing to letters that they selected themselves (see Fig. 5 for a graphical representation of the response shown in Supplementary Video 1).

This is not to deny that the assistant occasionally influenced participants. On the contrary, she sometimes redirected participants who seemed to lose their train of thought (as in Supplementary Video 3), requested clarification, interrupted, and said aloud a word before the participant finished spelling it. Importantly, however, this kind of influence is also common among speaking people, as when one interlocutor helps another find a word or completes their sentence. Indeed, influence is ubiquitous in communication because communication involves two or more individuals negotiating meaning together^41,42^. In light of the accuracy, speed, timing, and fixation data reported here, we do not consider instances of this kind of influence to be a reason to dismiss the ability of participants in our sample to convey their own thoughts.

We need to be clear about what we are not claiming. First, we studied a unique sample, comprising nonspeaking autistic people who were intentionally chosen because they were experienced letterboard users. We are not claiming that all nonspeaking autistic people can learn to convey their thoughts using a letterboard. Second, we have argued that a compelling reason to believe that participants in our study were spelling their own thoughts is their speed and visual fixation patterns. But we are not claiming that someone who spells slowly or whose eyes cannot be tracked is incapable of conveying their own thoughts. Additional research is needed to develop other methods and approaches to investigating communicative agency in nonspeaking people.

Even though our findings are limited to this unique sample of letterboard users, they are noteworthy because they cast doubt on the widely held belief among scientists and professionals that any nonspeaking autistic person who appears to communicate with assistance is actually responding to subtle cues from the assistant^14–17^. Our findings invite critical questions about what role the assistant serves, how the spelling skill is learned and by whom, and how technology can be leveraged to facilitate independence. They also suggest that some nonspeaking autistic people who communicate with assistance can indeed offer insight into their condition and lives.

Recent research has shown that the cognitive abilities of nonspeaking autistic people have been significantly underestimated^43^. Our study suggests that communication is another domain where conventional wisdom about nonspeaking autism requires revision.

## Methods

This study was approved by the University of Virginia Institutional Review Board for the Social and Behavioral Sciences (protocol number: 2389). The study was performed in accordance with relevant guidelines and regulations. Parents provided written informed consent for their children’s participation; participants additionally provided written informed consent or, if under 18 years of age, written assent. Consent to publish Supplementary Videos 1–3 was obtained from the relevant participants and their parents.

### Participants

Participants were recruited from a centre that specializes in supporting children and adults with limited speech to access other forms of communication. It also provides training and support to parents and caregivers. One of the communication methods taught at the centre involves the letterboard that is the subject of this study.

The centre identified 10 adolescents and young adults who had a diagnosis of autism, limited spontaneous speech, and at least two years of experience learning to use a letterboard. All were considered by staff members at the centre to be skilled letterboard users. The study was described to these 10 individuals and their parents; all agreed to participate. Participants did not receive compensation for their participation. Coders were unable to detect from video which letters one participant pointed to; there were therefore no usable data from this participant. At the time of their sessions, the remaining nine participants were, on average, 20.04 years old (*range*: 14.67 to 26.25 years; 1 female). Six participants were White, one was African American, one was Asian American, and one was Latinx. Supplementary Table S1 provides descriptive characteristics of the sample.

Parents provided documentation from a physician or clinical psychologist confirming their child’s autism diagnosis. Parents also completed a developmental history form and three standardized questionnaires: the Vineland Adaptive Behavior Scales 2^nd^ edition^44^, the Social Communication Questionnaire (SCQ)^45^, and the Social Responsiveness Scale-2^46^ (see Supplementary Methods for information about these questionnaires).

There is no consensus in the literature about how to define “nonspeaking,” “nonverbal,” or “minimally verbal”^47^, particularly for individuals over 17 years of age. The spoken language abilities of participants ranged from a few word approximations to a limited number of utterances or scripted phrases. Some participants could give verbal responses to highly practiced questions (e.g., “What’s your name?”), but none could respond verbally to open-ended questions of the type they were asked in this study (e.g., “Have you ever experienced uncertainty?”). On the parent-report SCQ^45^, all but one participant was reported to be able to speak using short phrases or sentences (item 1), but none was reported to be able to have a “to and fro” spoken conversation involving turn-taking or building on what a conversational partner had said earlier (item 2).

Parents reported seeking out letterboard instruction for their children because they were not able to communicate effectively using speech or other forms of augmentative and alternative communication despite having received, on average, 13.60 years (*range*: 10.42–21.75) of speech therapy in school and private practice prior to the introduction of the letterboard. Participants began learning to use the letterboard when they were, on average, 16.80 years old (*range*: 12.42–23.75). At the time of the study, they had, on average, 3.24 years (*range*: 2.25–5.83) of experience learning to use it. Eight of the nine participants were introduced to the letterboard at the centre where this study took place; one was introduced to it elsewhere. For details about how individuals are taught to use a letterboard at the centre where the study took place, see the Supplementary Methods.

At the time they participated in the study, the frequency with which participants visited the centre varied, from once every few months to 1–2 times per week. They participated in individual, parent/caregiver-child, and/or group settings. This variation reflected differences in how far away participants lived from the centre, other activities in which they were involved, their goals at a given point in time, scheduling constraints, and so on. All participants used the letterboard outside the centre (e.g., at home and school) with multiple trained assistants. In addition to the letterboard, all participants received instruction on other methods of communication, including speech, handwriting, and typing independently on a computer keyboard.

All participants had an Individualized Education Plan when they were in school; all had received applied behaviour analysis, the most common form of autism intervention, in school and privately for an average of 11.06 years (*range*: 5–18).

### Assistant

The same individual served as the assistant for all participants in this study. She was not compensated for her participation. She had over 25 years of experience working with individuals with complex communication profiles and had been providing instruction in the use of the letterboard as a means of augmentative and alternative communication for over four years at the start of this study. Although she was not the primary practitioner for all participants, she had interacted regularly with each for at least two years prior to data collection, in individual, parent/caregiver-child, and group settings at the centre where data were collected.

### Materials

*Head-mounted eye tracker*. Participants wore a Positive Science (www.positivescience.com) head-mounted eye-tracker. The eye-tracker had two small cameras mounted onto eyeglass frames: a scene camera and an eye camera. The scene camera was affixed above the right eye and pointed outward, capturing the scene in front of the participant (field of view: 63.01° horizontal x 50.13° vertical). The eye camera was attached to a flexible wire that extended out below the right eye and pointed toward it, capturing the participant’s eye movements. An infrared emitting diode was mounted on the same wire and illuminated the participant’s eye. The resolution of the two cameras was 30 frames per second. Video from the two cameras was fed by cables to a laptop on which the videos were recorded and stored.

*Letterboard*. The letterboard was a 21.6 × 27.9 cm laminated piece of paper with the following 32 items printed on it: 26 upper-case letters of the alphabet, 4 punctuation marks (comma, exclamation mark, question mark, period), and two icons (an X representing “delete” and a small bird representing “done”) (see Supplementary Methods for additional details about the letterboard).

### Procedure

Participants were seen individually for a session lasting about 30 minutes; the session was not part of any regularly scheduled appointment at the centre. They sat at a table in a small room at the centre where they regularly received instruction. A researcher explained how the eye-tracking glasses worked and gave participants the opportunity to handle them and to ask any questions. The researcher helped them put on the glasses, secured the glasses by tightening a strap behind the participant’s head, and adjusted the scene and eye cameras and infrared diode. Participants were encouraged to take breaks as needed; one participant did so.

The assistant sat to the right of participants. After asking a question (described below), she held the letterboard vertically in front of participants using her right hand. The letterboard was centred in front of participants’ right arm at a distance such that when participants pointed to letters, their arm remained bent slightly (viewing distance: ~40–55 cm; the letterboard subtended ~28–40° horizontal and ~22–30° vertical; individual letters subtended ~0.75–4° horizontal and ~2–3° vertical). Participants responded to questions by pointing to letters with the index finger of their right hand; one participant occasionally used the middle finger of the right hand.

*Calibration*. As described below, participants’ point of gaze was estimated from the scene and eye camera videos after the session, using Yarbus software (version 2.5.0, www.positivescience.com). To make those estimates, the software relied on calibration information collected at the beginning of each session in the form of clear and sustained looks to several locations on the letterboard (see Supplementary Methods for additional information about calibration).

*Lesson*. Two lessons were created to simulate a typical individual session at the centre. One lesson was about the corpse flower, and the other was about the Polynesians’ discovery of the Hawaiian Islands. Five participants were randomly assigned to receive one lesson, and four received the other. No participant had previously been exposed to the lesson they received or the questions they were asked.

After the initial calibration procedure, the assistant read aloud from the prepared text. Each lesson was broken into seven sections, and each section was two to six sentences long. After reading a section, the assistant asked participants between one and six questions related to the content of the previous section. There were four types of question: spelling (e.g., “Spell ‘decade’”); comprehension questions with one possible answer (e.g., “What [did I say] is the natural environment of the corpse flower?”); semi-open questions for which there were a limited number of possible answers (e.g., “What is another type of flower?”); and open-ended questions, for which there were an unlimited number of possible responses (e.g., “What’s something you’ve had to wait on?”). Each lesson consisted of 24 questions: The corpse flower lesson consisted of 7 spelling, 7 comprehension, 5 semi-open, and 5 open-ended questions; the corresponding numbers for the Hawaiian Islands lesson were 6, 7, 5, and 6. Because of experimenter error, responses from 23 rather than 24 questions were available from one participant.

### Video processing

After the session, the eye and scene videos were imported into Yarbus software (version 2.5.0, www.positivescience.com). Calibration points for each participant were marked on the appropriate frames of the scene camera video. The software then used estimates of the centre of the pupil and corneal reflection from the eye camera video to calculate the point of gaze within the scene camera video. The software created a new video (~30 frames/s) with a 1.25° radius red circle superimposed on each frame of the scene camera video (see Fig. 1). The location of this “gaze cursor” represented the participant’s point of gaze in that frame. This processed video was used for the frame-by-frame coding described next. For details about the spatial accuracy of the eye-tracking data, see the Supplementary Methods.

### Video coding

On average, the letterboard was available for responding on 17,401 frames (*range:* 14,224–21,255), or 9.67 minutes (*range*: 7.90–11.81) (see Supplementary Methods for details about when video coding of each response began). The processed scene camera video with the gaze cursor overlaid was coded, frame by frame, by two coders who used the open-source video coding software Datavyu (version 1.3.7, www.datavyu.org). One pair coded five participants; the other coded four participants. The primary coder in each pair coded 100% of each response from each assigned participant. The reliability coder in each pair coded a randomly selected 25% of each response from each assigned participant. The reliability coder for Pair 1 was also the primary coder for Pair 2. On each frame, coders indicated (a) which item (if any) a participant was pointing to, and (b) the location of the gaze cursor.

*Point coding*. Coders indicated that an item was being pointed to when a participant’s finger was in contact with that item on the letterboard. Practically, coders could tell from the rendered scene video when a point to an item began or ended because the letterboard would bow slightly at the point of contact as it began and would return to its unbowed position as the point ended. The primary and reliability coders in Pair 1 agreed on point coding on *M* = 97.3% of frames they both coded (*range* across participants: 95.2–98.9%); Cohen’s κ was *M* = 0.88 (*range*: 0.81–0.92). The primary and reliability coders in Pair 2 agreed on *M* = 97.2% of frames they both coded (*range*: 95.7–98.1%); Cohen’s κ was *M* = 0.90 (*range*: 0.84–0.94). Disagreements on two or more consecutive frames were resolved through discussion between the two coders; otherwise, the primary coder’s coding was used.

*Gaze coding*. The gaze cursor was visible, on average, on 91% (*range*: 86–100%) of the frames when the letterboard was available for responding (see Supplementary Table S3). It was not visible when participants blinked, looked outside the field of view captured by the scene camera, or when the software was unable to detect the pupil (see Supplementary Methods). On each frame when the gaze cursor was visible, coders indicated what item (if any) the gaze cursor overlapped. If the gaze cursor overlapped two adjacent items on the letterboard, coders indicated the item on which more of the gaze cursor overlapped; if it overlapped both items equally, they coded it as between the two. When the gaze cursor was on the letterboard but not overlapping any item, coders indicated that it was between items. When it was off the letterboard, coders indicated whether it was on the assistant or on something else in the room.

The primary and reliability coders in Pair 1 agreed on the location of the gaze cursor on *M* = 93.1% of frames they both coded (*range* across participants: 87.3–96.7%); Cohen’s κ was *M* = 0.93 (*range*: 0.87–0.97). The primary and reliability coders in Pair 2 agreed on *M* = 93.8% of frames they both coded (*range*: 92.8–96.1%); Cohen’s κ was *M* = 0.93 (*range*: 0.92–0.96). Disagreements on two or more consecutive frames were resolved through discussion between the two coders; otherwise, the primary coder’s coding was used.

## Supplementary information

Supplementary information.

Supplementary information 2.

Supplementary information 3.

Supplementary information 4.

## Data Availability

The data reported in this paper, along with the *R* code and workspace, are available at https://osf.io/jzdc6/.
