# Supplementary material for: Eye-tracking reveals agency in assisted autistic communication
Source: Sci Rep. 2020 May 12;10:7882. doi: 10.1038/s41598-020-64553-9 (PMC7217901; doi:10.1038/s41598-020-64553-9)
Supplement: Supplementary file 1 — Supplementary information. [file 41598_2020_64553_MOESM1_ESM.pdf]

Main text DOI: 10.1038/s41598-020-64553-9

Supplementary Information for

Eye-tracking reveals agency in assisted autistic communication

Vikram K. Jaswal*, Allison Wayne, & Hudson Golino

Department of Psychology, University of Virginia, Charlottesville, VA, USA.

*Correspondence to: jaswal@virginia.edu

This pdf file includes:

Captions for Supplementary Videos 1-3 ... 2
Supplementary Figures ... 3
Figure S1. Time to fixate correct letters as a function of letter position ... 3
Figure S2. Time to fixate the second letter in a bigram ... 4
Supplementary Tables ... 5
Table S1. Descriptive characteristics of participants ... 5
Table S2. Details of letters pointed to and words spelled ... 6
Table S3. Eye-tracking details for each participant ... 7
Table S4. Details of anticipatory fixations to correct letters ... 8
Supplementary Methods ... 9
Parent Questionnaires ... 9
Instruction on the Letterboard ... 9
Letterboard Details ... 10
Eye-tracking Calibration ... 11
Eye-tracking Spatial Accuracy ... 12
Video Coding ... 13
Supplementary Notes ... 15
General notes on linear mixed-effects regression models ... 15
IPI within vs. between words model ... 15
Predicting IPI from bigram distance and frequency ... 16
Fixation latency within vs. between words model ... 17
Predicting fixation latency from bigram distance and frequency ... 18
Supplementary Information References ... 20

Supplementary Video Captions

Supplementary Video 1 - Participant 2. This video shows Participant 2's response when asked "Can you think of something you have to wait for?" His response: "That is hard. (reset) I feel like world is waiting on me (done) (reset) not the other way around (done)". The eye-tracking video was produced using Yarbus software (version 2.5.0, www.positivescience.com).

Supplementary Video 2 - Participant 3. This video shows Participant 3's response when asked "Can you think of something you have to wait for?" His response: "Waiting for my dream girl (done)". The eye-tracking video was produced using Yarbus software (version 2.5.0, www.positivescience.com).

Supplementary Video 3 - Participant 9. This video shows Participant 9's response when asked "Have you ever experienced uncertainty?" Her response: "E(r)veryone does Im no different (done)". The participant calls out most letters as she points to them and says aloud words after spelling them. The eye-tracking video was produced using Yarbus software (version 2.5.0, www.positivescience.com).

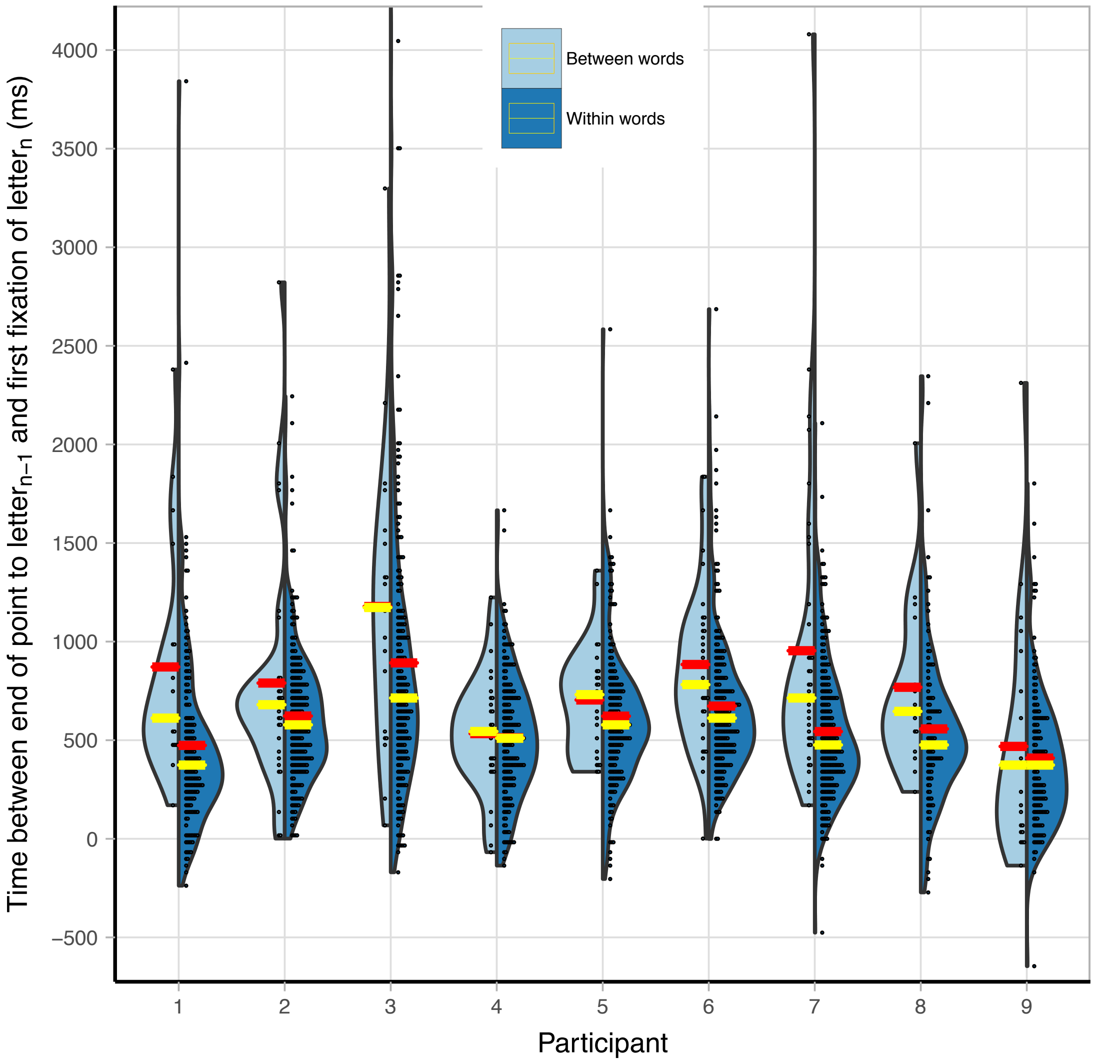

Figure S1. Time to fixate next correct letter in a response as a function of letter position. Time between end of point to correct letter$_{n-1}$ and first fixation of correct letter$_n$, as a function of whether the transition from letter$_{n-1}$ to letter$_n$ crossed a word boundary (light blue) or was within a word (dark blue). Each dot represents an individual datapoint. Yellow lines show the medians, and red lines show the means. Dots at or below 0 represent occasions where a participant's fixation of letter$_n$ began before or during the point to letter$_{n-1}$ and continued after the end of the point to letter$_{n-1}$. (0 of 230 between-word observations and 1 of 1664 within-word observations were slower than 4250 ms and are not shown.)

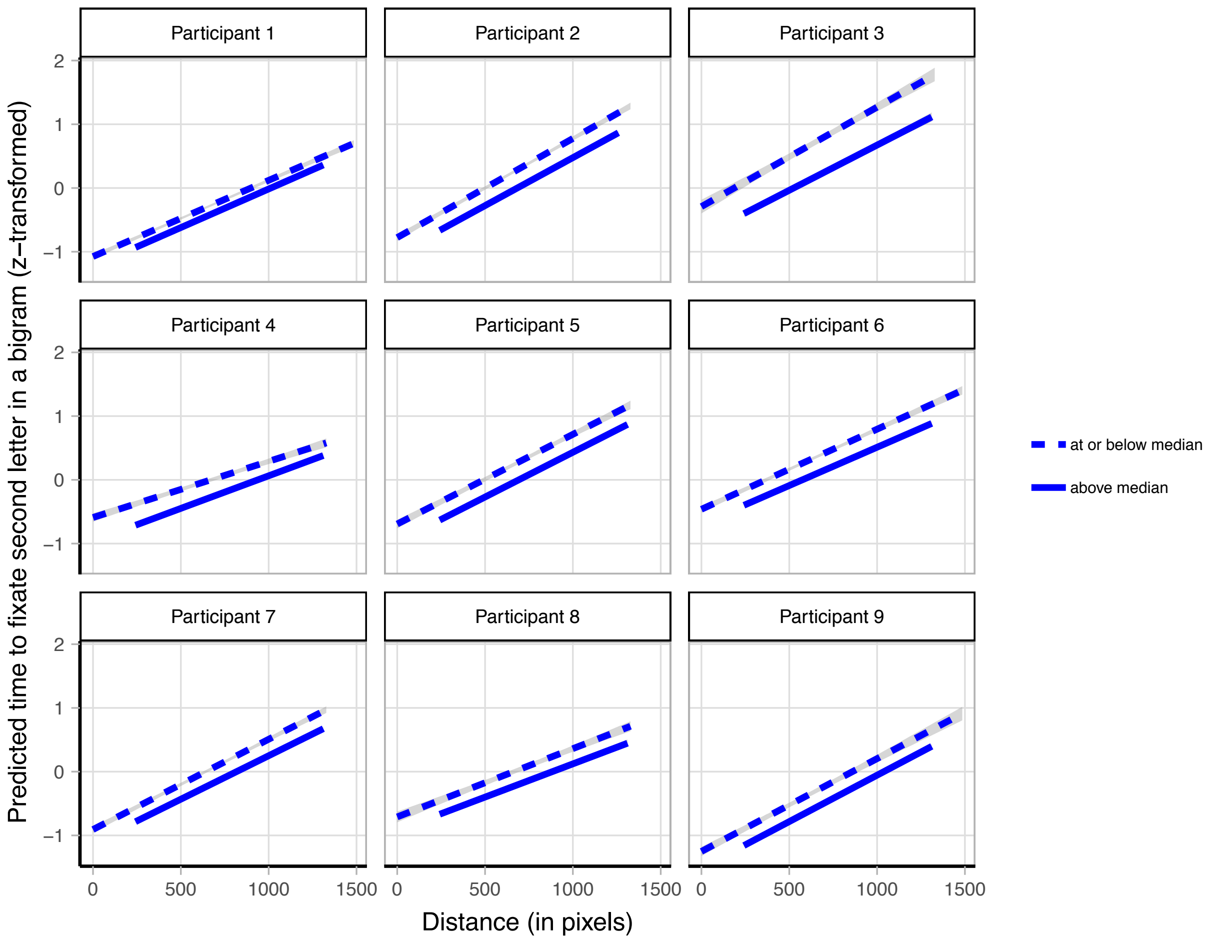

Figure S2. Time to fixate the second letter in a bigram as a function of bigram distance and frequency. Individual model fits for each participant predicting time between the end of the point to correct letter$_{n-1}$ and the first correct fixation of letter$_n$, using as predictors the distance between those letters on the letterboard and how frequently they occur consecutively in English. Bigram frequency is shown as a median split but was a continuous variable in the analysis reported in the text. Shading represents 95% confidence intervals.

Table S1. Descriptive characteristics of participants

	Age (years)	Sex	Years learning letter-board	Years in speech therapy before letterboard	Other common services (ages in years)			Standardized parent-report instruments		
					Applied behaviour analysis	Physical therapy	Occupational therapy	Vineland ABC Standard Score (%ile)	SCQ (Total)	SRS-2 (Raw Score)
1	19.75	M	2.75	12.00	2-14	3-6	5-current	68 (2%)	38	81
2	18.92	M	3.17	12.75	3-15	3-5	3-current	61 (0.5%)	32	66
3	26.25	M	2.50	21.75	4-22	None	2-current	38 (<0.1%)	32	100
4	21.92	M	3.92	13.00	5-10	None	3-current	64 (1%)	28	67
5	14.67	M	2.25	10.42	2-current	2-5	None	56 (0.2%)	24	85
6	17.42	M	2.50	13.42	1.5-14	None	1.5-14	36 (<0.1%)	31	81
7	20.83	M	3.92	14.92	4-10	None	4-18	64 (1%)	28	89
8	18.00	M	2.33	10.67	Yes*	None	5-17	68 (2%)	30	98
9	22.58	F	5.83	13.75	7-18	None	3-current	53 (0.1%)	36	71

Vineland ABC = Vineland Adaptive Behavior Composite from the Vineland Adaptive Behavior Scales, Second Edition[44]. SCQ = Social Communication Questionnaire-Lifetime[45]; individuals with total scores above 15 (along with clinical judgment) are recommended for additional autism evaluation. SRS-2 = Social Responsiveness Scale-2nd Edition, parent report[46]; raw scores above 70 (along with clinical judgment) are recommended for additional autism evaluation. * = Participant 8 participated in applied behaviour analysis, but ages were not provided.

Table S2. Details of letters pointed to and words spelled

	Word accuracy			Letter accuracy			
	# words attempted	# words spelled correctly	% correctly spelled words	# letters needed to spell all attempted words correctly	Total # letters pointed to	# correct letters pointed to*	% correct points
1	78	67	86%	395	417	395	95%
2	66	61	92%	324	340	324	95%
3	44	37	84%	269	277	269	97%
4	79	61	77%	375	425	375	88%
5	55	41	75%	272	288	270	94%
6	81	74	91%	397	412	396	96%
7	80	68	85%	379	393	377	96%
8	84	76	90%	411	435	410	94%
9	72	51	71%	353	396	352	89%

* When "# correct letters pointed to" is less than "# letters needed to spell all attempted words correctly," the participant misspelled one or more words by omitting a letter.

Table S3. Eye-tracking details for each participant

	% of response time gaze cursor visible	On letterboard		Off letterboard		Spatial accuracy
		On items	Between items	Something else in room	Assistant	
1	94% (11.11/11.80)	79.9%	17.4%	2.6%	0.0%	2.76°
2	89% (7.02/7.90)	73.0%	25.8%	1.3%	0.0%	0.82°
3	86% (9.84/11.42)	82.8%	14.3%	1.2%	1.7%	0.57°
4	93% (9.09/9.73)	71.6%	22.5%	5.9%	0.0%	2.41°
5	96% (7.67/8.00)	87.9%	10.9%	0.8%	0.4%	1.26°
6	86% (7.91/9.16)	81.7%	14.0%	4.3%	0.0%	1.79°
7	87% (8.65/9.91)	83.0%	16.8%	0.2%	0.0%	3.48°
8	87% (8.95/10.29)	67.1%	24.6%	8.4%	0.0%	7.21°
9	100% (8.78/8.79)	85.2%	13.6%	1.0%	0.3%	3.60°

% of response time gaze cursor visible = time the letterboard was available for responding and the gaze cursor visible during the session as a percentage of the time the letterboard was available for responding (ratios show the times in minutes). On letterboard vs. off letterboard = the percentage of time the gaze cursor was coded as on the letterboard (on items on the letterboard or in the space between items on the letterboard) or off the letterboard (on something else in the room or on the assistant). Spatial accuracy = average distance (in degrees of visual angle) between centre of gaze cursor and centre of calibration targets (lower number represents better spatial accuracy) (see Supplementary Methods online for how spatial accuracy was calculated).

Table S4. Details of anticipatory fixations to correct letters

	% anticipatory fixations	Serial position of anticipatory fixations			
		1st	2nd	3rd	> 3rd
1	55% (218/395)	34% (75/218)	32% (70/218)	16% (34/218)	18% (39/218)
2	88% (284/324)	30% (86/284)	34% (95/284)	24% (68/284)	12% (35/284)
3	99% (267/269)	25% (66/267)	26% (70/267)	20% (54/267)	29% (77/267)
4	68% (255/375)	26% (65/255)	33% (85/255)	21% (54/255)	20% (51/255)
5	92% (248/270)	17% (42/248)	31% (76/248)	30% (74/248)	23% (56/248)
6	70% (276/396)	30% (83/276)	33% (91/276)	24% (65/276)	13% (37/276)
7	80% (303/377)	27% (81/303)	33% (100/303)	23% (70/303)	17% (52/303)
8	32% (133/410)	31% (41/133)	32% (43/133)	20% (27/133)	17% (22/133)
9	59% (208/352)	35% (72/208)	28% (59/208)	20% (42/208)	17% (35/208)

% anticipatory fixations = percentage of correct letters that were fixated for 99 ms or more before they were pointed to. Serial position = percentage of anticipatory fixations of correct letters that were the first, second, third, or higher fixation of correct $letter_n$ on the letterboard after the point to $letter_{n-1}$ had ended. (Ratios shown in parentheses.)

Supplementary Methods

Parent Questionnaires

Vineland Adaptive Behavior Scales, 2nd Edition[44]. The VABS is a standardized parent questionnaire, which provides scores in three domains: Communication, Daily Living Skills, and Socialization. Together, these three domain scores yield an Adaptive Behavior Composite standard score.

Social Communication Questionnaire, Lifetime[45]. The SCQ is a standardized 40-item parent-report measure of the child's autistic symptomatology in social interaction, communication, and repetitive behaviour. When used as a screening instrument, an individual who scores 15 or above (along with clinical judgment) is recommended to be referred for further evaluation. The SCQ demonstrates good agreement with "gold standard" diagnostic measures like the Autism Diagnostic Observation Schedule[48].

Social Responsiveness Scale 2, parent report[46]. The SRS-2 is a standardized 65-item instrument measuring social challenges in autism in 5 domains: social awareness, social cognition, social communication, social motivation, and restricted interests and repetitive behaviour. An individual with a raw score of 70 or above (along with clinical judgment) is recommended to be referred for further evaluation.

Instruction on the Letterboard

Instruction on the letterboard is personalized to each user. In a typical session at the centre where the study took place, the instructor reads aloud a piece of age-appropriate text, pausing to spell aloud and define words that may be unfamiliar to students. After each passage, the instructor asks students to spell a word, recall a piece of information from the text, answer

closed-ended questions, or (at later stages) synthesize the material or offer an opinion. The instructor holds the letterboard vertically in front of the student, who responds by pointing to letters with the index finger of their dominant hand.

At initial stages of training, the focus is on practicing to point deliberately and accurately, which many students find difficult given their sensory motor challenges[11]. Thus, early in training, the instructor asks questions with known answers and provides verbal coaching for motor planning, encouraging students to lift their arms, for example, and to extend their finger. The instructor does not physically touch the student at any point in the training. As students become proficient in pointing to letters, verbal coaching is faded, and the instructor increases the complexity of the instruction and adds open-ended questions. Advanced instruction focuses on training the skills needed to be able to communicate without an assistant holding the letterboard; independent communication is the ultimate goal. How quickly students progress depends on their initial skill, the degree of sensory motor involvement, how much and how consistently they are able to practice, the skill of the assistants with whom they practice, and so on—the same kinds of factors that affect the acquisition of other complex skills, like learning to play a sport or an instrument.

Letterboard Details

The letters and punctuation on the letterboard appeared as white characters in Arialle 80-point font on a brown background, and the delete and done icons (a small bird) were orange (the word "done" was superimposed on the bird in white). Each letter of the alphabet was 2 cm tall; their maximum width varied depending on the letter, from 0.5 cm for the "I" to 2.67 cm for the "W." The 26 letters were arranged alphabetically in 5 rows; the first four rows contained 5

letters, and the last row contained 6. Because letters varied in width, the distance from the edge of one letter to the closest point of a horizontally adjacent letter varied from 1.5-3.5 cm. Rows were separated from each other by 2 cm. The four punctuation marks, delete, and done icons appeared in a column to the right of the alphabet matrix; responses to these items were not analysed in this study. The opposite side of the letterboard included numbers and mathematical symbols; one response for four of the participants began on the number side of the letterboard, but the number part of that response was not analysed.

Eye-tracking Calibration

The assistant began each session by holding the letterboard in front of participants and asking them to deliberately and carefully look at and point to several items (*M*: 10.4 items, *range:* 5-22). These items were distributed across the letterboard and included four items at or near the corners (A, E/delete, U/V, and Z/done) and items in the centre row (e.g., M, K, O). All participants pointed correctly to the requested items. The minimum number of calibration points requested was five, but a researcher watching the video feed requested additional points (or repetition of some points) if participants blinked, squinted, or failed to maintain a sustained look to a requested item as they pointed to it, or if the letterboard shifted out of view of the scene video as the calibration procedure took place.

To provide additional potential calibration points, participants were additionally asked to deliberately and carefully look at and point to the letters in the first name of the assistant; three were further asked to look at and point to the letters in their own first name. All participants correctly spelled the requested names.

Four participants repeated the calibration procedure again after their session had started: One who removed the glasses to take a break, and three whose eye and/or scene camera videos were reported by the researcher watching the video feed to have shifted during the session so that they were no longer capturing the intended image. This shift occurred when the glasses were bumped or slipped, or the angle at which the letterboard was held by the assistant had changed from where it was held during the initial calibration. In these cases, the session was stopped, the cameras were adjusted, and the calibration procedure repeated.

Eye-tracking Spatial Accuracy

We estimated the spatial accuracy of the eye-tracking data for each participant from the calibration procedure. Following the protocol established in previous work using a similar head-mounted eye tracking system[49], we selected 20 frames for each participant where they had been instructed to look at and point to particular targets during the calibration procedure (five consecutive frames from immediately before a participant pointed to each of four different targets at the corners of the letterboard). For each frame, we calculated the distance in pixels between the centre of the gaze cursor in the processed video (representing the Yarbus software's estimate of the participant's point of gaze; version 2.5.0, www.positivescience.com) and the centre of the calibration target, and then converted the distances to degrees of visual angle. (For the four participants who repeated the calibration procedure after their session had begun, we averaged the two spatial accuracy scores.) A smaller value represents better spatial accuracy. The average spatial accuracy was 2.66° (*range*: 0.57-7.21°) (see Supplementary Table S3).

Spatial accuracy can be affected by a number of factors, including individual differences in eye colour, eyelash thickness and direction, eyelid closure, eye physiology; moisture or tears;

how anxious participants are; the lighting of the room; and so on[50]. Furthermore, spatial accuracy was not uniform across the letterboard for a given participant, and so it was not possible to apply a fixed adjustment to a participant's data. Participants in eye-tracking studies are often excluded if the calibration procedure yields poor spatial accuracy during the calibration procedure[51]. We chose to report eye-tracking data from all nine participants, including those whose calibration yielded relatively poor spatial accuracy, recognizing that for some participants it represents an underestimate of how likely they were to fixate a target letter before touching it.

Video Coding

The processed scene camera video with the gaze cursor overlaid was coded using the open-source video coding software Datavyu (version 1.3.7, www.datavyu.org). Coding of a response began when the assistant placed the letterboard in front of the participant, on the first frame of the video on which the letters were in focus. Coding of a response ended when a participant pointed to the "done" icon for the final time in a response or, if a participant did not point to "done" at the end of a response, when the assistant began removing the letterboard from the participant's field of view. Some responses were interrupted briefly when the assistant removed the letterboard from participants' field of view to write down part of a response, for example, or when she was directed by the researcher watching the video feed to adjust the position of the letterboard so it would be captured fully within the scene camera. The average number of these "resets" in a session was 9.22 (*range*: 2-18). When a reset occurred, coding was suspended at the end of the previous point and resumed on the first frame when the letterboard was replaced and the letters were in focus in the video.

Gaze coding. It was not possible to code the location of the gaze cursor on every frame the letterboard was available for responding for three reasons: 1) participants sometimes blinked (*M*: 1,399 frames; *range*: 0-2,367); 2) their point of gaze was sometimes outside the view captured by the scene camera (*M*: 170 frames; *range*: 2-311); or 3) their pupil occasionally was not detected by the Yarbus eye-tracking software (version 2.5.0, www.positivescience.com) even though the eye was open (*M*: 30 frames; *range*: 0-259).

Note that some letters on the letterboard were narrower than others (e.g., I vs. W). As a result, the gaze cursor had to be closer to the horizontal centre of some letters than others in order for coders to indicate that a look to that letter had occurred. A different coding scheme could have involved creating equally sized areas of interest for the letterboard matrix such that a look to the letter "I," for example, would be coded if the gaze cursor were in that letter's area of interest even if the gaze cursor was not touching the letter itself. We used the more conservative coding approach, requiring the gaze cursor to actually be touching part of a letter for coders to indicate it as a look to that letter.

Supplementary Notes

General notes on linear mixed-effects regression models

Inter-point interval (IPI) and fixation latency variables were transformed to normal distributions using the orderNorm algorithm in the *bestNormalize* package[52] in *R*. To account for the nesting of our data (observations within participants), we used the *lme4*[53] and *lmerTest* packages[54]. We kept the random effects structure as maximal[55], allowing both slope and intercept to vary by participant. We calculated pseudo-R^2 for the fixed effects, using the *MuMIn* package[56]. For analyses involving bigram distance and frequency variables, those two variables were on different scales, and so were grand mean centred, with standardized betas reported.

IPI within vs. between words model

To investigate whether participants were slower to point to the first letter of words in a multi-word response than letters within words (Fig. 3a), we conducted a linear mixed-effects regression analysis on the inter-point interval (IPI) to correct letters. We compared a null model to one with letter position (within a word vs. the first letter of a new word) as a fixed factor: IPI ~ 1 + (1 | participants) vs. IPI ~ letter.position + (1 + letter.position | participants). The logLikelihood of the null model with three degrees of freedom was -3649. The logLikelihood of the final model with letter position added and six degrees of freedom was -3532, which represents a significant increase in goodness of fit, χ^2 (3 df) = 233.09, $p < .0001$. AIC and BIC statistics decreased from the null to the final model, also indicating an increase in goodness of fit (AIC: 7304 to 7077; BIC: 7322 to 7112). The pseudo-$R^2_{\mathrm{GLMM(m)}}$ of the fixed effect was 0.06. Number of observations: 2767; Groups: Participants, 9.

Predicting IPI from bigram distance and frequency

To measure the distance between letters within bigrams on the letterboard, we used an electronic image of the letterboard and calculated the Euclidean distance (in pixels) between the centres of each pair of letters. To obtain bigram frequencies, we used log-transformed bigram frequency counts for directly adjacent lowercase letters within a word calculated from full-text articles from three months of the *New York Times* (~14 million words)[35]. Because these bigram frequency counts were generated from adjacent letters within words, we excluded IPIs that crossed word boundaries.

To investigate whether bigram frequency predicted inter-point interval (IPI) above and beyond the distance between the two letters in a bigram, we conducted a linear mixed-effects regression on the IPI between consecutive correct letters within words. We first compared a null model without any fixed factors to one with bigram distance as a fixed factor: IPI ~ 1 + (1 | participants) vs. IPI ~ distance + (1 + distance | participants). Our final model added bigram frequency as a second fixed factor: IPI ~ distance + frequency + (1 + distance + frequency | participants). Figure 3b shows predictions from the final model for each participant.

The logLikelihood of the null model with three degrees of freedom was -3152. The logLikelihood of the second model with bigram distance added and six degrees of freedom was -2968, which represents a significant increase in goodness of fit, χ^2 (3 df) = 368.00, $p < .0001$. The loglikelihood of the final model with both bigram distance and bigram frequency and 10 degrees of freedom was -2904, which represents a significant increase in goodness of fit over the model with bigram distance alone, χ^2 (4 df) = 128.37, $p < .0001$ AIC and BIC statistics decreased from the null to the final model, also indicating an increase in goodness of fit (AIC: 6309 to 5947 to 5827; BIC: 6327 to 5982 to 5885). The pseudo-$R^2_{GLMM(m)}$ for fixed effects increased from 0.10

in the model with just bigram distance to 0.13 in the final model with both bigram distance and frequency. Number of observations: 2422; Groups: Participants, 9.

As expected, in the final model, the distance between two letters in a bigram predicted IPI: The farther the first letter was from the second on the letterboard, the slower participants were to point to the second letter, $\beta = 0.32$, SE = 0.05, $t(9.11) = 6.81$, $p < .0001$, 95% CI [0.21, 0.42]. This is not surprising given that it takes longer to move one's finger a greater distance compared to a shorter one. But as explained in the main text, bigram frequency predicted IPI above and beyond distance: The more frequent a bigram was, the faster participants were to point to the second letter in the bigram, $\beta = -0.18$, SE = 0.02, $t(8.66) = 9.25$, $p < .0001$, 95% CI [-0.23, -0.14].

Fixation latency within vs. between words model

To investigate whether participants were slower to fixate the first letter of words in a multi-word response than letters within words (Fig. S1), we conducted a linear mixed-effects regression analysis on the time between the end of a point to $letter_{n-1}$ and the first correct fixation of $letter_n$. We compared a null model to one with letter position (within a word vs. the first letter of a new word) as a fixed factor: fixation.latency ~ 1 + (1 | participants) vs. fixation.latency ~ letter.position + (1 + letter.position | participants). The logLikelihood of the null model with three degrees of freedom was -2624. The logLikelihood of the final model with letter position added and six degrees of freedom was -2604, which represents a significant increase in goodness of fit, χ^2 (3 df) = 39.97, $p < .0001$. AIC and BIC statistics decreased from the null to the final model, also indicating an increase in goodness of fit (AIC: 5254 to 5220; BIC: 5271 to 5253).

The pseudo-$R^2_{GLMM(m)}$ of the fixed effect was 0.02. Number of observations: 1894; Groups: Participants, 9.

Predicting fixation latency from bigram distance and frequency

To investigate whether bigram frequency predicted latency to fixate letters above and beyond the distance between the two letters on the letterboard, we conducted an analysis similar to the one predicting IPI from bigram distance and frequency described above, but we used fixation latency as the dependent variable instead. Specifically, we conducted a linear mixed-effects regression on the time between the end of the point to correct $letter_{n-1}$ and the first correct fixation of $letter_n$ within words. We first compared a null model without any fixed factors to one with bigram distance as a fixed factor: fixation.latency ~ 1 + (1 | participants) vs. fixation.latency ~ distance + (1 + distance | participants). Our final model added bigram frequency as a second fixed factor: fixation.latency ~ distance + frequency + (1 + distance + frequency | participants). Figure S2 shows predictions from the final model for each participant.

The logLikelihood of the null model with three degrees of freedom was -2305. The logLikelihood of the second model with bigram distance added and six degrees of freedom was -2131, which represents a significant increase in goodness of fit, χ^2 (3 df) = 348.1, $p < .0001$. The loglikelihood of the final model with both bigram distance and bigram frequency and 10 degrees of freedom was -2087, which represents a significant increase in goodness of fit over the model with bigram distance alone, χ^2 (4 df) = 87.96, $p < .0001$ AIC and BIC statistics decreased from the null to the final model, also indicating an increase in goodness of fit (AIC: 4615 to 4273 to 4193; BIC: 4631 to 4306 to 4247). The pseudo-$R^2_{GLMM(m)}$ for fixed effects increased from 0.17 in

the model with just bigram distance to 0.20 in the final model with both bigram distance and frequency. Number of observations: 1664; Groups: Participants, 9.

As expected, in the final model, the distance between two letters in a bigram predicted fixation latency: The farther the first letter was from the second on the letterboard, the slower participants were to fixate the second letter after the point to the first letter had ended, $\beta = 0.40$, SE = 0.03, $t(7.81) = 12.53$, $p < .0001$, 95% CI [0.33, 0.47]. But as explained in the main text, bigram frequency predicted fixation latency above and beyond distance: The more frequent a bigram was, the faster participants were to fixate the second letter in the bigram, $\beta = -0.19$, SE = 0.04, $t(8.71) = 5.13$, $p = .0007$, 95% CI [-0.27, -0.11].

Supplementary Information References

(Numbering continues from References in main text)

48. Corsello, C., Hus, V., Pickles, A., Risi, S., Cook, E. H., Leventhal, B. L. & Lord, C. Between a ROC and a hard place: Decision making and making decisions about using the SCQ. *J. Child Psychol. Psychiatry* **48,** 932-940 (2007).

49. Franchak, J. M., Kretch, K. S. & Adolph, K. E. See and be seen: Infant-caregiver social looking during locomotor free play. *Dev. Sci.* **21,** e12626 (2018).

50. Nyström, M., Andersson, R., Holmqvist, K. & van de Weijer, J. The influence of calibration method and eye physiology on eyetracking data quality. *Behav. Res. Methods* **45**, 272-288 (2013).

51. Dalrymple, K. A., Manner, M. D., Harmelink, K. A., Teska, E. P., Elison, J. T. An examination of recording accuracy and precision from eye tracking data from toddlerhood to adulthood. *Front. Psychol.* **9,** 803 (2018).

52. Peterson, R. A. & Cavanaugh, J. E. Ordered quantile normalization: a semiparametric transformation built for the cross-validation era. *J. of App. Stat.* 10.1080/02664763.2019.1630372 (2019).

53. Bates, D., Machler, M., Bolker, B. & Walker, S. Fitting linear mixed-effects models using lme4. *J. Stat. Softw.* **67,** 1-48 (2015).

54. Kuznetsova, A., Brockhoff, P. B. & Christensen, R. H. B. lmerTest package: Tests in linear mixed effects models. *J. Stat. Software* **82,** 1-26 (2017).

55. Barr, D. J., Levy, R., Scheepers, C. & Tily, H. J. Random effects structure for confirmatory hypothesis testing: Keep it maximal. *J. Mem. Lang.* **68,** 255-278 (2013).

56. Bartoń, K. *MuMIn: Multi-Model Inference* (R package version 1.43.6, https://CRAN.R-project.org/package=MuMIn, 2019).
